# Supplementary material for: Inferring causal connectivity from pairwise recordings and optogenetics
Source: PLoS Comput Biol. 2023 Nov 7;19(11):e1011574. doi: 10.1371/journal.pcbi.1011574 (PMC10656035; doi:10.1371/journal.pcbi.1011574)
Supplement: S1 Appendix — The following proofs follow the book from Brady Neal [65]. Text A. Identification proof of ATE with linear parametric form of IV. Text B. Non-parametric identification of CACE with potential outcomes. (PDF) [file pcbi.1011574.s001.pdf]

Supporting information

S1 Appendix The following proofs follow the book from Brady Neal [1].

Text A. Identification proof of ATE with linear parametric form of IV The simplest identification proof of the IV estimator is obtained by assuming a linear parametric form $Y = \beta X + U$, and $X = \alpha(1 - Z) + E$. Remember that the OLS estimate of β is rubbish since $\operatorname{cov}(X, U) \neq 0$. We start by considering the effect of the instrument on the outcome $\mathrm{E}[Y(Z=0) - Y(Z=1)]$, which, if computed directly, would give a combination of α and β, while we are interested in β. Using the IV assumptions, we obtain

$$
\begin{aligned}
&\mathrm{E}[Y|Z=0] - \mathrm{E}[Y|Z=1] \\
&= \mathrm{E}[\beta X + U|Z=0] - \mathrm{E}[\beta X + U|Z=1], \text{(ii) Exclusion Restriction} \\
&= \beta(\mathrm{E}[X|Z=0] - \mathrm{E}[X|Z=1]) + \mathrm{E}[U] - \mathrm{E}[U], \text{(iii) Instrumental Independence}
\end{aligned}
$$

From this, we can uncover the IV estimator

$$\beta = \frac{\mathrm{E}[Y|Z=0] - \mathrm{E}[Y|Z=1]}{\mathrm{E}[X|Z=0] - \mathrm{E}[X|Z=1]}$$

Note the denominator differs from zero because of (i) Relevance. However, the linear parametric assumptions are not satisfying. First, the assumption requires homogeneity, i.e., the treatment effect is the same for every trial. Moreover, it assumes a constant effect from X on Y, which is not true if, for example, Y is refractory. Moreover, the assumed constant effect of Z on X does not hold since when $Z = 0$, the stimulus-response of X can vary. The next section shows that the IV estimand has a more satisfying non-parametric interpretation but not of the ATE.

Text B. Non-parametric identification of CACE with potential outcomes In the following non-parametric identification, we use assumption (iv) monotonicity. Using a non-parametric form, we do not obtain the true ATE, but a local ATE, also known as the compliers average causal effect (CACE). To obtain the CACE estimate we stratify stimulus response X given Z in four groups,

"compliers" : $X_i(Z=0) = 1, X_i(Z=1) = 0$,

"deniers" : $X_i(Z=0) = 0, X_i(Z=1) = 1$,

"always takers" : $X_i(Z=0) = 1, X_i(Z=1) = 1$,

"never takers" : $X_i(Z=0) = 0, X_i(Z=1) = 0$.

Of course, these groups are not identifiable since we cannot determine two different responses in one trial.

By assuming that there are no "deniers" we get the monotonicity assumption $X_i(Z=0) \geq X_i(Z=1)\, \forall\, i$. Again, the index represents potential outcomes; imagine a world where we had two versions of X_i in the same trial i, one where $Z = 0$ and one where $Z = 1$. Moreover, it is assumed that the net effect of "always takers" and "never takers" will be zero in Y. More formally this translates for "never takers" to: $\mathrm{E}[Y(X=0) - Y(X=1)|X(Z=0) = 0, X(Z=1) = 0] = 0$. Given these assumptions, we can derive the CACE estimator **??**. In the following notation, we have reduced the

arguments on the right-hand side for $Y(Z=z)$ to $Y(z)$ and similarly for X in lack of space.

$$
\begin{aligned}
&\mathrm{E}\left[Y(Z=0)-Y(Z=1)\right] \\
&\quad= \mathrm{E}\left[Y(0)-Y(1)|X(0)=1,X(1)=0\right]P(X(0)=1,X(1)=0), \text{ “compliers”.} \\
&\quad+ \mathrm{E}\left[Y(0)-Y(1)|X(0)=0,X(1)=1\right]P(X(0)=0,X(1)=1), \text{ “deniers”.} \\
&\quad+ \mathrm{E}\left[Y(0)-Y(1)|X(0)=1,X(1)=1\right]P(X(0)=1,X(1)=1), \text{ “always takers”.} \\
&\quad+ \mathrm{E}\left[Y(0)-Y(1)|X(0)=0,X(1)=0\right]P(X(0)=0,X(1)=0), \text{ “never takers”.}
\end{aligned}
$$

Here we have used the law of total expectation $\mathrm{E}[Y]=\sum_i \mathrm{E}[Y|X_i]\,P(X_i)$. With the above assumptions for “deniers”, “always takers” and “never takers” the right-hand side is reduced to only compliers.

$$
\begin{aligned}
&\mathrm{E}\left[Y(Z=0)-Y(Z=1)\right] \\
&\quad= \mathrm{E}\left[Y(0)-Y(1)|X(0)=1,X(1)=0\right]P(X(0)=1,X(1)=0), \text{ “compliers”.}
\end{aligned}
$$

By rearranging, we obtain

$$
\mathrm{E}\left[Y(Z=0)-Y(Z=1)|X(Z=0)=1,X(Z=1)=0\right]=\frac{\mathrm{E}\left[Y(Z=0)-Y(Z=1)\right]}{P(X(Z=0)=1,X(Z=1)=0)}.
$$

Moreover, since we are only dealing with “compliers”, trials where the pre-synaptic neuron responds to stimuli if it has not spiked before stimuli onset and vice versa, we can write

$$
\begin{aligned}
&\mathrm{E}\left[Y(Z=0)-Y(Z=1)|X(Z=0)=1,X(Z=1)=0\right] \\
&\quad= \mathrm{E}\left[Y(X=0)-Y(X=1)|X(Z=0)=1,X(Z=1)=0\right]
\end{aligned}
$$

We now apply assumption (iii) Instrumental Independence to obtain $\mathrm{E}\left[Y(Z=0)-Y(Z=1)\right]=\mathrm{E}\left[Y|Z=0\right]-\mathrm{E}\left[Y|Z=1\right]$. Moreover, the probability of being a “complier”, $P(X(0)=1,X(1)=0)$, is given by the probability of not being an “always taker” or “never taker” as the probability of being a “defier” is zero due to monotonicity. This can be stated as

$$
\begin{aligned}
P(X(Z=0)=1,X(Z=1)=0) &= 1-P(X=0|Z=0)-P(X=1|Z=1) \\
&= 1-(1-P(X=1|Z=0))-P(X=1|Z=1) \\
&= \mathrm{E}\left[X|Z=0\right]-\mathrm{E}\left[X|Z=1\right].
\end{aligned}
$$

Using this relation we obtain the non-parametric IV estimator

$$
\mathrm{E}\left[Y(X=0)-Y(X=1)|X(Z=0)=1,X(Z=1)=0\right]=\frac{\mathrm{E}\left[Y|Z=0\right]-\mathrm{E}\left[Y|Z=1\right]}{\mathrm{E}\left[X|Z=0\right]-\mathrm{E}\left[X|Z=1\right]}.
$$

With this identification, we can now interpret the IV estimator as CACE

$$
\beta=\mathrm{E}\left[Y(X=0)-Y(X=1)|X(Z=0)=1,X(Z=1)=0\right]
$$

In other words, an estimator of the post-synaptic response to trials where the pre-synaptic neuron responds to stimuli if it has not spiked before stimuli onset and vice versa, which is exactly what we are interested in.

References

1. Neal B. Introduction to Causal Inference. 2015;.
