# Supplementary material for: Inferring causal connectivity from pairwise recordings and optogenetics
Source: PLoS Comput Biol. 2023 Nov 7;19(11):e1011574. doi: 10.1371/journal.pcbi.1011574 (PMC10656035; doi:10.1371/journal.pcbi.1011574)
Supplement: Attachment — Submitted filename: Causal connectomics plos biology 2023 response to reviewers.pdf [file pcbi.1011574.s002.pdf]

Reviewer #1: The authors suggest the use of the idea of Instrumental Variables from economics to facilitate the inference of synaptic connectivity (effective connectivity?) from neural data where optogenetics can be employed as a perturbation. The analysis of the proposed method is done on simulated neural data showing the positive effect it has on the inference process.

I find the general idea of this paper quite interesting and important. Using perturbation (optogenetics or otherwise) as a tool for facilitating inference of connectivity is a very promising tool. Also, the concept of instrumental variables is a welcome novel addition to efforts for inferring connectivity. However, I find the results themselves weak and the exposition of the results quite unclear.

We appreciate that the reviewer find the general idea important and we have changed the manuscript according to the the reviewer's insightful comments and suggestions.
Regarding the results, my criticism are as follows

-the authors perform their analysis on simulated data. Although the authors do study the effect of various features of the simulated network (such as type external field), important features are unexplored. For example the network size is rather small (200 neurons: 100 excitatory, 100 inhibitory), the connectivity is stated to be excitatory. I would have excepted the simulations to be done with larger networks, and in particular with more realistic model neurons, e.g. Hodgkin-Huxely or Integrate-and-Fire. There many biologically realistic neural network models, e.g. of the visual cortex, and the authors come from one of the major centres for building such models. So I am a bit surprised that such analyses are not performed.

We acknowledge that our analysis was performed on simulated data with a network size of up to 500 neurons, which may be considered small compared to biologically realistic neural networks. However, we believe that it is crucial to understand the dynamics and characteristics of smaller networks before progressing to larger and more complex ones. As we have found in our study, the size of the network influences the condition number, and our results are well-suited for the range presented.

Moreover, we recognize the value of using more realistic neuron models, such as the Hodgkin-Huxley or Integrate-and-Fire models. However, our focus in this study was to understand the fundamental aspects of relatively simple networks. We found that even with simpler models, results shows that common methods give substantial errors which the proposed IV,DiD method improves providing valuable insights. In our view, the validation of the proposed methods poses a significant challenge, given the complexity and variability of neural networks in vivo. In this context, the use of simple models to test, compare, and verify the applicability is important for the following reasons:

Foundation Building: Simple models serve as a foundational basis for understanding the fundamental principles underlying neural connectivity and the performance of inference methods. By starting with a simplified network, we can isolate and study the effects of individual parameters and variables for connectivity reconstruction. This foundational understanding is essential before progressing to more complex and biologically realistic models.

Computational Efficiency: Complex neural network models often involve a large number of neurons, intricate connectivity patterns, and detailed biophysical properties, leading to high computational demands. Simple models, on the other hand, are computationally more tractable, allowing for a more exhaustive exploration of the parameter space, especially considering convergence and a thorough evaluation of the performance of different inference methods.

Control Over Ground Truth: In simple models, the ground truth effective connectivity is known a priori, enabling a direct comparison. This is often not possible with real neural data or highly complex models, where the ground truth effective connectivity is unknown or uncertain.

Noise and Confound Control: Simple models allow for the controlled introduction of different types and levels of noise and confounds, facilitating the systematic evaluation of the robustness and sensitivity of inference methods under various conditions. This is crucial for understanding the limitations of different methods and for developing strategies to mitigate the effects of noise and confounds.

Facilitating Comparison: The use of simple models provides a common ground for comparing different inference methods. Since the same simple model can be used by different researchers and research groups, it facilitates the comparison of results across studies and the establishment of benchmarks for the performance of different methods.

In conclusion, while it is ultimately necessary to test and validate inference methods on biologically realistic neural network models and real neural data, the use of simple models is a crucial and necessary step in development and validation. Simple models provide a controlled and computationally tractable environment for understanding the fundamental principles, limitations, and potential improvements of different inference methods, laying the groundwork for their application to more complex and realistic scenarios.

Extending the manuscript to include more complex models would make it considerably longer and more intricate. Instead, we intend to explore these more realistic situations in subsequent studies.

We understand the reviewer's expectation for analyses involving larger networks and more realistic model neurons. However, we believe that our current approach provides a solid foundation for understanding basic network dynamics, which is essential before delving into more complex scenarios. We sincerely appreciate the reviewer's suggestions and will certainly consider them in our future research endeavors.

- The results are section 2.4 are based on stimulating 5 neurons. How does this change if the analysis is performed on a larger fraction of neurons being stimulated? Is it important to know a priori what the fraction of activated neurons are? if so, how can one have a good estimate of that in a real data?

Thank you for your question regarding the impact of stimulating a larger fraction of neurons on our analysis. As shown in Figure 4b, the error increases as a larger fraction of neurons are stimulated. This suggests that the fewer neurons stimulated, the better the results. Therefore, while it is not essential to know a priori the exact number of neurons that will be

activated, our analysis indicates that stimulating a smaller number of neurons yields more accurate results.

In real data, estimating the fraction of activated neurons can be challenging. However, our findings suggest that it is beneficial to stimulate as few neurons as possible to minimize error. This insight can be valuable when designing experiments or interpreting real data, even if it is not possible to precisely determine the fraction of activated neurons beforehand. To make this point more clear we have discussed the proposition of making opsins as local as possible recited here for the reviewers benefit.

"The main problem with optogenetic stimulation, when used to infer connectivity, is its non-local property. This is due to the inverse relationship between changes in light intensity, and the affected number of neurons combined with a logarithmic relation between light intensity and photocurrent \cite{wang2007high}. In addition, the distribution of membrane potentials across neurons is relatively flat \citep{destexhe1999impact,rudolph2006use,pare1998impact}, making neurons highly sensitive to perturbations. One could imagine situations where optogenetic activation was more local. If, for example, the membrane potential distributions were skewed with the mode far from the threshold, a powerful stimulus would be required for a neuron to elicit spikes. There could also be other ways of making optogenetic stimulation more local. For example, if one engineered opsins or brain tissue that are more light absorbent (e.g. by ubiquitously producing melanin), one could stimulate more locally. Having melanin under a ubiquitously expressed promotor in the brain would dramatically make optogenetics more local and could probably be a target for the construction of transgenic animals. How to engineer more localized stimulation is a significant problem when causally interrogating a system."

- The authors do not explore their approach on any real dataset. It is true that for real datasets, the ground truth is not known. Still one can compare the results of different methods and see if they indeed do give different results, and how big the difference is.

We appreciate the reviewer's suggestion to compare our approach with different methods using real datasets, despite the ground truth being unknown. We agree that this would be a valuable analysis to perform. However, as we have chosen to focus on smaller and less biophysically detailed neural networks in this manuscript, we believe that including an analysis on real datasets would be beyond the scope of the current study. Nonetheless, we recognize the importance of this analysis and plan to include it in a future manuscript, where we will explore more realistic neural networks and compare our approach with other methods using real datasets. Thank you for this constructive suggestion.

Regarding the presentation, although I really liked the introduction, I found the presentation of the Results right after the Introduction without having the author know the main methodological approaches made the paper difficult to read. The Methods section at the end is also very cluttered and jumps between definitions, proofs and material (e.g. 4.3.2) that can also be described in the results section. Some of the material (e.g. proofs) can be moved to a supplemental information/apprendices. I think the paper needs a major rewrite even if not further analyses are described.

We sincerely appreciate the reviewer's feedback on the presentation of the manuscript. We understand the concern about the placement of the Results section immediately after the

Introduction, and the cluttered nature of the Methods section. Therefore we did our best effort to follow your suggestion for a major rewrite carefully. See the changes pdf for details, in summary we have

- Moved the introduction to Instrumental variables previously found before the proofs in the methodology section to the results section to clarify the beginning of the results.
- Moved the proofs of identification to the supporting information section, to reduce the clutteredness of the methods
- Included details for approximating a realistic optogenetic perturbation in the results section, to reduce the clutteredness of the methods
- Formatted some text in the methods section, to reduce the clutteredness of the methods

Reviewer #2: This study is interesting and creative, but unlike the authors I would not discount the usefulness of two-photon optogenetic stimulation of arbitrary subgroups of cells in vivo (Packer et al. 2015 https://www.nature.com/articles/nmeth.3217) to infer functional connectivity – it creates far more favourable and realistic conditions than blanket 1-photon optogenetic stimulation, and can already be applied to hundreds of presynaptic and thousands of postsynaptic neurons across brain areas (Fisek et al. 2023 https://www.nature.com/articles/s41586-023-06007-6) and more in the future. It would be useful to compare blanket 1-photon and patterned 2-photon stimulation when inferring functional connectivity.

We appreciate the reviewer's recognition of the creativity and interest of our study. We also acknowledge the potential advantages of two-photon optogenetic stimulation of arbitrary subgroups of cells in vivo, as highlighted in the referenced papers by Packer et al. (2015) and Fisek et al. (2023). We agree that this technique creates more favorable and realistic conditions. However, we would like to clarify that we do not intend to discount the usefulness of two-photon optogenetic stimulation. Our intention is to highlight some of its current limitations, such as the inability to use it in freely moving animals or across different animal species due to the requirement of head fixation. While these limitations may be addressed in the future, they currently pose significant challenges that restrict the use of this technology in certain contexts. Therefore, we believe it is important to continue exploring and improving other approaches, such as 1-photon methods, which may be more suitable for certain applications.

To clarify this view we have added the following paragraph in the discussion:

"*On the other hand, we acknowledge the potential advantages of two-photon optogenetic stimulation of arbitrary subgroups of cells \textit{in vivo}; e.g. \cite{packer2014simultaneous}. This technique can in some cases give more favorable and realistic conditions compared to blanket 1-photon optogenetic stimulation and holds the potential for application to a vast number of presynaptic and postsynaptic neurons across various brain regions \citep{Fisek2023}. Our intention is therefore not to undermine the utility of two-photon optogenetic stimulation. Rather, we aim to underscore some of its existing limitations, such as its inapplicability in freely moving animals or across different animal species for example due to the necessity for head fixation. Although limitations may be surmounted in the future, they currently present considerable challenges that constrain the applicability of this technology in specific contexts. Consequently, we posit the necessity to persist in the*

exploration and enhancement of alternative approaches, such as 1-photon methods, which may be more apt for certain applications. This perspective underscores the necessity for a multifaceted approach to technological advancement in neuroscience, recognizing the strengths and weaknesses of each method to optimize their application in diverse research contexts.”

My main concern, however, is that the simulated model system (eq. 2) used by the authors to apply their statistical methods to may not exhibit sufficiently realistic types of noise that are found in cortical networks in vivo, leading to an underestimation of confounds. London et al. (2010) showed that cortical networks are chaotic because small perturbations in the spiking history grow. This is because the probability of a presynaptic spike evoking an extra postsynaptic spike (which is on the order df a percent) multiplied by the fan-out of the presynaptic neuron (the number of its postsynaptic targets, which in the cortex is on the order of thousands) is much larger than one. Two spiking histories of the same network in response to the same stimulus will therefore be massively different if looked at in small time bins, which may interfere with the statistical analysis methods presented in this manuscript. Are the networks used in this manuscript sufficiently chaotic, and do perturbations in these models grow as fast (e.g. with a gain of 28, London et al. 2010) as they do in vivo? The authors should ensure and demonstrate that their model system generates sufficiently realistic irreproducibility of spiking histories of the network.

We appreciate the reviewer's concern about the realism of the noise in our simulated model system and its potential impact on the estimation of confounds. We acknowledge that the chaotic nature of cortical networks, as described by London et al. (2010), may interfere with the statistical analysis methods presented in our manuscript.

In our model, there is intrinsic noise in the network in the form of Bernoulli trials on the log probability of the neural states. Following the formula from London et al. (2010), which involves the average fan-out multiplied by the effective connectivity, we observe the effect of reducing sparsity in Figure 4a. For the strongest connected neurons and zero sparsity, this value would be about 10 (200 * 0.05). Although this does not reach the gain reported by London et al. (2010), we observe sufficient trends for comparing the proposed inference methods. As the gain increases, the IV,DiD method proves to be more robust. However, larger networks adversely affect all proposed methods, albeit to a lesser extent for the IV,DiD method (Figure 5).

We have intentionally focused on simple networks in this manuscript to understand the limitations of these methods. As we note in the discussion, to move towards more realistic scenarios, we need to further investigate how to improve this methodology, for example to properly model the latency distribution. We recognize that the IV,DiD method is a promising starting point, but acknowledge that there is much work to be done to ensure robustness in sufficiently realistic neural networks.

We sincerely appreciate the reviewer's insightful comments and will take them into consideration as we continue to refine and improve our methodology.
